# 16S rRNA Sequencing and Metabolomics to Analyze Correlation Between Fecal Flora and Metabolites of Squabs and Parent Pigeons

**DOI:** 10.3390/ani15010074

**Published:** 2025-01-01

**Authors:** Xiaobin Li, Shengchen Zheng, Haiying Li, Jiajia Liu, Fan Yang, Xiaoyu Zhao, Yafei Liang

**Affiliations:** 1 College of Animal Science, Xinjiang Agricultural University, Urumqi 830091, China; lxb262819@163.com (X.L.); 13565573464@163.com (S.Z.); yangfan312au@163.com (F.Y.); vz_zxy@163.com (X.Z.); 13934057294@163.com (Y.L.); 2 Moyu Blue Sea Pigeon Industry Co., Ltd., Hetian 848101, China; liujiajia861018@163.com

**Keywords:** squabs, parent pigeons, feces, bacterial diversity, metabolomics

## Abstract

Intestinal microorganisms are essential for maintaining homeostasis, health, and development, playing a critical role in nutrient digestion, growth, and exercise performance in pigeons. In young pigeons, the gut microbiota is primarily acquired through pigeon milk, meaning the microbial composition of parent pigeons directly influences microbial colonization in squabs. However, research on the correlation between the gut microbial diversity of parent pigeons and their offspring remains scarce. This study investigates the fecal microbiota and metabolites of 10 pairs of parent pigeons and 20 squabs raised under a 2 + 2 system. Fecal samples were collected at 15 days of age, and differences in the microbiota and metabolites between the two groups were analyzed using 16S rRNA sequencing and LC-MS/MS. The results showed the following: (1) Squabs exhibited significantly lower α diversity, with a reduction in their Chao1 index and observed OTUs compared to the parent pigeons. (2) Firmicutes dominated the fecal microbiota in both groups, but parent pigeon feces showed a notably higher abundance of Proteobacteria. At the family level, 10 distinct families were identified, with 9 at the genus level and 4 at the species level. (3) A LEfSe analysis identified 16 significantly different bacterial species in the parent pigeons and 7 in the squabs. Functional gene abundance was highest in the metabolism, genetic information processing, and environmental information processing pathways. (4) An LC-MS/MS analysis in cationic mode identified 218 metabolites, with 139 upregulated and 79 downregulated in the squabs relative to the parents. These metabolites were primarily concentrated in five functional categories and enriched in 33 pathways, 2 of which showed significant differences. In conclusion, significant differences in both the α and β diversity of fecal microbiota were observed between squabs and parent pigeons, with similar bacterial species but marked differences in abundance. Metabolite analysis revealed greater richness in the parent pigeon feces. These findings suggest that future gut modulation using beneficial bacteria, such as probiotics, could potentially enhance host health based on microbial and metabolite compositions.

## Simple Summary

Intestinal microorganisms are critical for the health and development of pigeons, influencing nutrient digestion and exercise performance. This study examines the gut microbiota of squabs and their parent pigeons, with a particular focus on the effect of parental microbiota on offspring. Ten pairs of parent pigeons and twenty squabs were analyzed, with fecal samples collected at 15 days of age. The results revealed that squabs exhibited significantly lower α diversity than their parents, with Firmicutes predominating in both groups but a higher abundance of Proteobacteria found in the parental feces. Distinct bacterial species were identified, along with significant differences in the functional genes associated with metabolism. LC-MS/MS analysis identified 218 metabolites, revealing notable discrepancies between the squabs and parents. These findings indicate substantial differences in gut microbiota compositions and metabolites, suggesting the potential for probiotic interventions to improve gut health in pigeons.

## 1. Introduction

The colonization of gastrointestinal microorganisms is essential for the health of both livestock and poultry. Previous studies have demonstrated that gut microbiota diversity in early life stages positively influences intestinal health, immunity, and overall growth in young livestock and poultry [1]. However, the colonization patterns of intestinal microbes differ between young poultry (such as chickens, ducks, and geese) and domestic animals (including horses, cattle, sheep, and pigs), primarily due to variations in dietary sources [2,3]. Unlike chicks, ducklings, and goslings, squabs receive nutrition through the pigeon milk secreted by their parents [4,5]. This milk not only satisfies the nutritional needs of squabs but also introduces microorganisms into their gastrointestinal tract during feeding [6]. Consequently, the early microbial colonization in the gastrointestinal tract of squabs is influenced by both pigeon milk and the surrounding environment [7]. The microbial structure, composition, and diversity in the gut of parent pigeons, along with their gastrointestinal health, significantly impact microbial colonization and intestinal health in their offspring [8].

The pigeon industry in China has experienced steady growth, positioning pigeons as the fourth largest poultry product after chickens, ducks, and geese [9]. As pigeon breeding becomes increasingly important to national production, squab health plays a critical role in advancing China’s pigeon industry. Squabs, being altricial birds, rely entirely on pigeon milk for nutrition after hatching, as they are incapable of self-feeding. This milk, secreted by the parent’s crop and fed mouth-to-mouth [10], is essential for their growth and development. Factors influencing squab development include growth characteristics, crop milk quality, and the nutritional management of breeding pigeons [11]. Prior research, including our own, has shown that pigeon milk is a thick paste, typically white or pale yellow, with a rancid odor [12]. It is nutrient-rich, containing protein, fat, minerals, fatty acids, and amino acids, and includes desquamated epithelial cells believed to contribute specifically to squab development [13]. Studies also highlight that pigeon milk is rich in immunoglobulin A (IgA), immunoglobulin G (IgG), acid phosphatase (ACP), and alkaline phosphatase (ALP), providing substantial maternal antibodies [14]. Additionally, pigeon milk contains a diverse microbiota, with microorganisms forming a complex symbiotic relationship with their host. The initial microbiome can have a lifelong impact on the host [15]. Research suggests that, similar to mammalian milk, pigeon crop milk aids in establishing the microbiota, metabolic regulation, and immune system development in offspring [16]. Recent studies indicate that microbiota diversity in the pigeon crop is more complex than that in the small intestine or rectum, with *Lactobacillus* being the dominant genus [9].

To further elucidate the differences and similarities in intestinal microbiota between squabs and parent pigeons, this study focused on 15-day-old squabs. Fecal samples were collected from both squabs and parent pigeons, all housed in identical feeding environments. 16S rRNA sequencing and a metabolomics analysis were performed to identify variations in fecal microbiota and metabolites. This study aimed to investigate the factors underlying the differences in intestinal microbiomes between squabs and parent pigeons, offering insights into the early stages of microbiome colonization in pigeons.

## 2. Materials and Methods

### 2.1. Ethical Considerations

All animal care and handling procedures in this study were conducted in accordance with the Guidelines for the Care and Use of Laboratory Animals in China and were approved by the Animal Care Committee of Xinjiang Agricultural University, China (protocol permit number: 2020024).

### 2.2. Animal and Experimental Design

The research was conducted at Blue Sea Pigeon Industry Co., Ltd., located in Moyu County, Xinjiang, China. Tarim pigeons, bred under the 2 + 2 model, were selected for this study. In a controlled environment with identical feeding and dietary conditions, 10 pairs of healthy parent pigeons and 20 squabs were chosen. The parent pigeons were on their second clutch, with each pair raising two squabs. The experiment was divided into two groups: the squab group and the parent group. Fecal samples were collected from both groups at 15 days of age. Throughout the experiment, all parent pigeons had ad libitum access to feed and water.

### 2.3. Sample Collection

On day 15, fecal samples were collected from squabs and parent pigeons in the early morning before feeding. Veterinarians from Blue Sea Pigeon Industry Co., Ltd., assisted with the collection process. Prior to sampling, the anal areas of both the squabs and parent pigeons were disinfected with 75% medical alcohol. Following disinfection, acupressure was applied to stimulate defecation. Feces from each pair of parent pigeons were pooled into a single parent sample, and feces from the two squabs of the same parents were pooled into one squab sample. The samples were then placed in sterile, enzyme-free, RNA-free cryovials and immediately frozen in liquid nitrogen. In total, 10 mixed parent samples and 10 mixed squab samples were collected for analysis.

### 2.4. Sample Determination

#### 2.4.1. Fecal Microbiological Assay

##### Sample Quality Control

For sample quality control, the amplicon generation process targeted 16S rRNA/18S rRNA/ITS genes from distinct regions (16SV4, 16SV3, 16SV3-V4, 16SV4-V5, 18SV4, 18SV9, ITS1, ITS2, ArcV4) using specific primers (e.g., 16SV4: 515F-806R, 18SV4: 528F-706R, 18SV9: 1380F-1510R) with barcodes. PCR reactions were carried out with 15 µL of Phusion^®^ High-Fidelity PCR Master Mix (New England Biolabs, Ipswich, MA, USA), 0.2 µM of forward and reverse primers, and approximately 10 ng of template DNA. Thermal cycling conditions included initial denaturation at 98 °C for 1 min, followed by 30 cycles of denaturation at 98 °C for 10 s, annealing at 50 °C for 30 s, elongation at 72 °C for 30 s, and final elongation at 72 °C for 5 min.

##### PCR Product Quantification and Qualification

The PCR products were purified using magnetic bead purification. The samples were mixed in uneven density ratios based on the concentration of the PCR products. After thorough mixing, the PCR products were detected, and the target bands were recovered.

##### Library Preparation and Sequencing

Sequencing libraries were generated, and indices were added. The library was checked for quantification using Qubit and real-time PCR, and size distribution was assessed with a BioAnalyzer. The quantified libraries were pooled based on the effective library concentration and required data amount and then sequenced on Illumina platforms.

#### 2.4.2. Fecal Metabolomics Analysis

##### Sample Processing

The fecal samples (100 mg) were individually ground with liquid nitrogen, and the homogenate was resuspended in pre-chilled 80% methanol using vortex mixing. The samples were incubated on ice for 5 min, followed by centrifugation at 15,000× *g* at 4 °C for 20 min. A portion of the supernatant was diluted with LC-MS-grade water to achieve a final concentration of 53% methanol. The samples were then transferred to fresh Eppendorf tubes and centrifuged again at 15,000× *g* at 4 °C for 20 min. Finally, the supernatant was injected into the LC-MS/MS system for analysis.

UHPLC-MS/MS analyses were performed using a Vanquish UHPLC system (Thermo Fisher, Bremen, Germany) coupled with an Orbitrap Q ExactiveTM HF mass spectrometer or Orbitrap Q ExactiveTM HF-X mass spectrometer (Thermo Fisher, Germany) at Novogene Co., Ltd. (Beijing, China). The samples were injected onto a Hypersil Gold column (100 × 2.1 mm, 1.9 µm) and analyzed using a 12 min linear gradient at a flow rate of 0.2 mL/min. The eluents for both the positive and negative polarity modes were eluent A (0.1% FA in water) and eluent B (methanol). The solvent gradient was as follows: 2% B for 1.5 min; 2–85% B for 3 min; 85–100% B for 10 min; A linear gradient from 100% B to 2% B was applied over 10.1 min; and 2% B at 12 min. The Q ExactiveTM HF mass spectrometer was operated in positive/negative polarity mode with a spray voltage of 3.5 kV, a capillary temperature of 320 °C, a sheath gas flow rate of 35 psi, an aux gas flow rate of 10 L/min, and an S-lens RF level of 60, with an aux gas heater temperature of 350 °C.

The raw data files generated by UHPLC-MS/MS were processed using CompoundDiscoverer 3.3 (CD3.3, ThermoFisher) for peak alignment, peak picking, and the quantification of each metabolite. The key parameters were set as follows: peak area correction was performed with the first QC, mass tolerance was set to 5 ppm, signal intensity tolerance was set to 30%, and a minimum intensity threshold was applied. Subsequently, the peak intensities were normalized to the total spectral intensity. The normalized data were used to predict molecular formulas based on additive ions, molecular ion peaks, and fragment ions. The peaks were then matched against the mzCloud (https://www.mzcloud.org/ Accessed on 19 March 2024), mzVault, and MassList databases to obtain accurate qualitative and relative quantitative results. Statistical analyses were conducted using R (version R-3.4.3), Python (version 2.7.6), and CentOS (release 6.6). When the data were not normally distributed, they were standardized using the following formula: sample raw quantitation value/(sum of sample metabolite quantitation values/sum of QC1 sample metabolite quantitation values). This yielded the relative peak areas. Compounds with a CV of relative peak areas greater than 30% in the QC samples were excluded, and the final metabolites’ identification and relative quantification results were obtained.

### 2.5. Statistical Analysis

Statistical analysis was performed using the Student’s *t*-test or one-way analysis of variance (ANOVA). Data normality was assessed with the Shapiro–Wilk test, and the homogeneity of variance was evaluated using Levene’s test. Parametric tests were only applied to datasets meeting these assumptions. For ANOVA, Tukey’s Honestly Significant Difference (HSD) test was used for post hoc pairwise comparisons when significant differences (*p* < 0.05) were detected. All statistical analyses were conducted using SPSS software (version 27, IBM Corp., Armonk, NY, USA), with statistical significance set at *p* < 0.05.

Metabolite identification was carried out using the KEGG (https://www.genome.jp/kegg/pathway.html Accessed on 19 March 2024), HMDB (https://hmdb.ca/metabolites Accessed on 19 March 2024), and LIPIDMaps (http://www.lipidmaps.org/ Accessed on 19 March 2024) databases. Principal component analysis (PCA) and partial least squares discriminant analysis (PLS-DA) were performed using MetaX. A univariate analysis (*t*-test) was employed to determine statistical significance (*p*-value). Differential metabolites were identified as those with VIP > 1, *p*-value < 0.05, and fold change ≥ 2 or ≤0.5. Volcano plots, based on log2(FoldChange) and −log10(*p*-value), were generated using ggplot2 in R. For clustering heatmaps, differential metabolite intensities were normalized using z-scores, and the plots were created using the Pheatmap package in R. A correlation analysis of the differential metabolites was performed using the cor() function in R (method = Pearson), with statistically significant correlations determined by cor.mtest() (*p*-value < 0.05). Correlation plots were generated using the corrplot package in R. The functions and metabolic pathways of the metabolites were analyzed using the KEGG database. The enrichment of the metabolic pathways was determined when the ratio (x/n > y/N) was met, and pathways with *p*-value < 0.05 were considered statistically significantly enriched.

## 3. Results

### 3.1. Microbial Diversity and Composition

#### 3.1.1. OTU-Based Wayne Graph Analysis and UPGMA Clustering Tree

**Figure 1:**
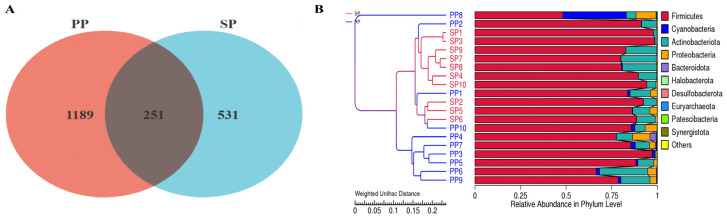
OTU-based Wayne graph analysis and UPGMA clustering tree. (**A**): Wayne diagram analysis; (**B**): UPGMA clustering tree based on OTUs.

To compare the fecal microbiota between the parent pigeons (PP group) and squabs (SP group), a Venn diagram analysis based on Operational Taxonomic Units (OTUs) and Unweighted Pair Group Method with Arithmetic Mean (UPGMA) clustering was conducted. As shown in Figure 1A, the Venn diagram revealed 251 shared OTUs between the SP and PP groups, indicating a common set of species and highlighting a fundamental similarity in their microbiota. However, the SP group contained 531 unique species, while the PP group had 1189 unique species, underscoring significant differences in microbial composition between the two groups. The UPGMA clustering tree further demonstrated that the fecal microbiota of the SP and PP groups form distinct clusters, reinforcing the independence and diversity of their microbiota profiles.

#### 3.1.2. Analysis of Bacterial Alpha Diversity in Feces of Pigeon Parent (PP) Group and Pigeon Squab (SP) Group

To further explore the microbial differences between parent pigeons and squabs, the bacterial Alpha diversity was analyzed, with its results summarized in Table 1. Alpha diversity indices, including Chao1, dominance, goods_coverage, observed_otus, pielou_e, Shannon, and Simpson, were used to assess diversity levels. The Chao1 and observed_otus indices in the PP group were significantly higher than those in the SP group (*p* < 0.05), indicating greater species richness in the PP group. This increased bacterial diversity may provide the PP group with a wider array of metabolic pathways and enzymatic functions, facilitating the metabolism of a broader range of both exogenous and endogenous compounds and promoting the synthesis of a more diverse spectrum of metabolites.

#### 3.1.3. Principal Component (PCA) Analysis and Principal Coordinates (PCoA) Analysis of SP and PP Groups

**Figure 2:**
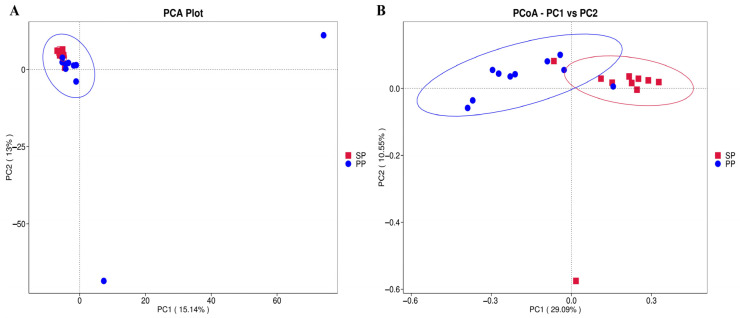
Principal component analysis (PCA) and principal coordinate analysis (PCoA). (**A**): PCA; (**B**): PCoA.

A principal component analysis (PCA) and principal coordinate analysis (PCoA) were utilized to visually assess the microbiota similarities and differences between the SP and PP groups. As shown in Figure 2A, the first principal component (*x*-axis) explains 15.14% of the variation, while the second principal component (*y*-axis) accounts for 13.04%. Although some differences in bacterial composition between the PP and SP groups are observed, their microbiota exhibit a substantial degree of similarity. PCoA further refines this by providing a clearer representation of the bacterial composition distribution. In Figure 2B, the first principal coordinate (*x*-axis) accounts for 29.09% of the variation, with the second coordinate (*y*-axis) explaining 10.55%. These results align with the PCA results, reinforcing the similarity in bacterial composition between the two groups and confirming the close relationship of their microbiota.

#### 3.1.4. Effects of Relative Abundance of Fecal Flora in SP Group and PP Group

**Figure 3:**
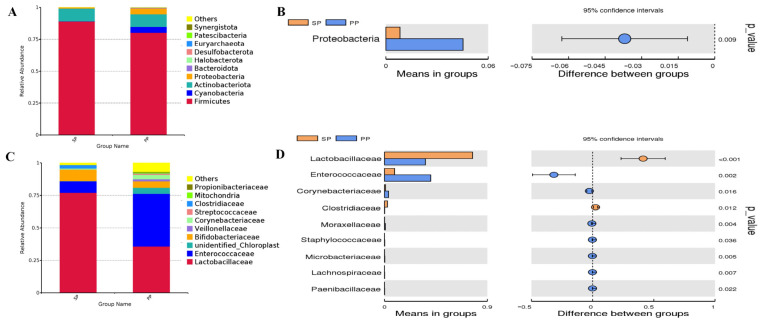

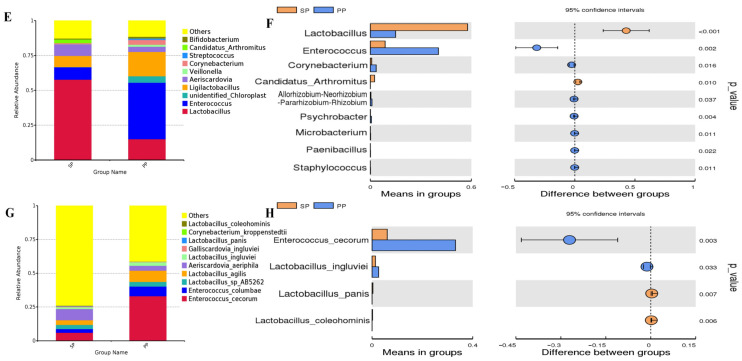
Effects of the relative abundance of the fecal flora. (**A**,**C**,**E**,**G**): Influence of the relative abundance of the fecal flora at the phylum, family, genus, and species levels; (**B**,**D**,**F**,**H**): *T*-test for the effects of the relative abundance of the fecal flora at the phylum, family, genus, and species levels.

Following the PCA and PCoA analyses, a more detailed examination of the fecal microbiota composition between the SP and PP groups was performed by evaluating changes in relative abundance at various taxonomic levels (Figure 3). As shown in Figure 3A,B, the top ten dominant phyla in both groups are identical, ranked by abundance as Firmicutes, Cyanobacteria, Actinobacteriota, Proteobacteria, Bacteroidota, Halobacterota, Desulfobacterota, Euryarchaeota, Patescibacteria, and Synergistota. Together, these phyla comprise over 99.97% of the total microbial community. A notable difference is observed in the abundance of Proteobacteria, which significantly varies between the SP and PP groups (15.47% vs. 84.53%, *p* < 0.01). Certain species of Proteobacteria play a role in nutrient absorption, metabolite production, and microbial interactions, suggesting that this discrepancy may reflect differences in nutrient utilization and metabolic regulation between squabs and parent pigeons. As shown in Figure 3C,D, the dominant bacterial groups in both the SP and PP groups account for over 93.19%, predominantly consisting of Lactobacillaceae and Enterococcaceae. In Figure 3E,F, the primary fecal bacteria in both groups represent more than 87.15%, with *Lactobacillus* and *Enterococcus* being the most abundant. Figure 3G,H highlight key bacterial species, accounting for over 26.00% in both groups, including *Enterococcus cecorum*, various *Lactobacillus* species, and *Corynebacterium kroppenstedtii*.

#### 3.1.5. Effect of LefSe Analysis of Fecal Flora in SP Group and PP Group

**Figure 4:**
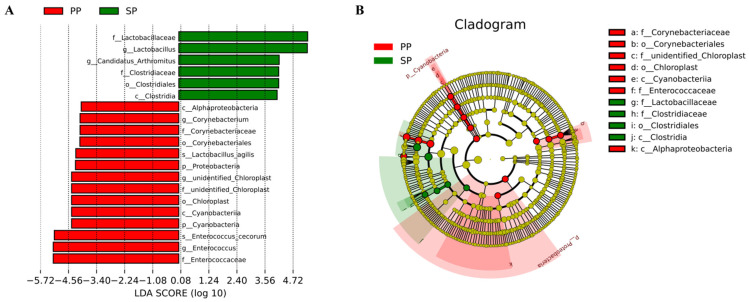
Significant differences were assessed using Linear Discriminant Analysis Effect Size (LEfSe), with a linear discriminant analysis score > 4 and a *p*-value < 0.05. (**A**) Histogram of LDA scores showing significant bacterial taxa that differ between the PP (red) and SP (green) groups; (**B**) Cladogram showing the phylogenetic relationships of significantly different taxa between the PP (red) and SP (green) groups.

To precisely identify key differential bacteria between the SP and PP groups, a LefSe analysis of fecal microbiota was conducted. Figure 4 illustrates 24 statistically significant biomarkers distinguishing the two groups. In the SP group, a distinct set of biomarkers, particularly f_Lactobacillaceae and g_Lactobacillus, was prevalent. These taxa are typically associated with probiotic effects, supporting the development of the intestinal tract and early immune system in squabs. In contrast, the PP group displayed different biomarkers, likely reflecting the mature and stable intestinal environment of parent pigeons. Notably, p_Proteobacteria exhibited significant differences in the PP group, indicating its critical role in nutrient absorption, immune regulation, and other physiological functions in parent pigeons.

#### 3.1.6. Functional Prediction of Tax4Fun in Feces of SP Group and PP Group

**Figure 5:**
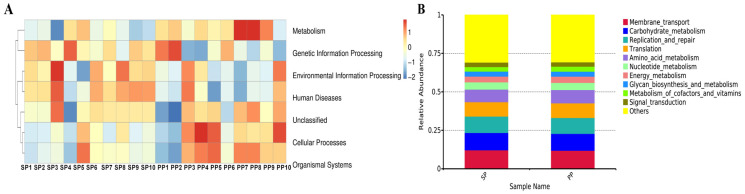

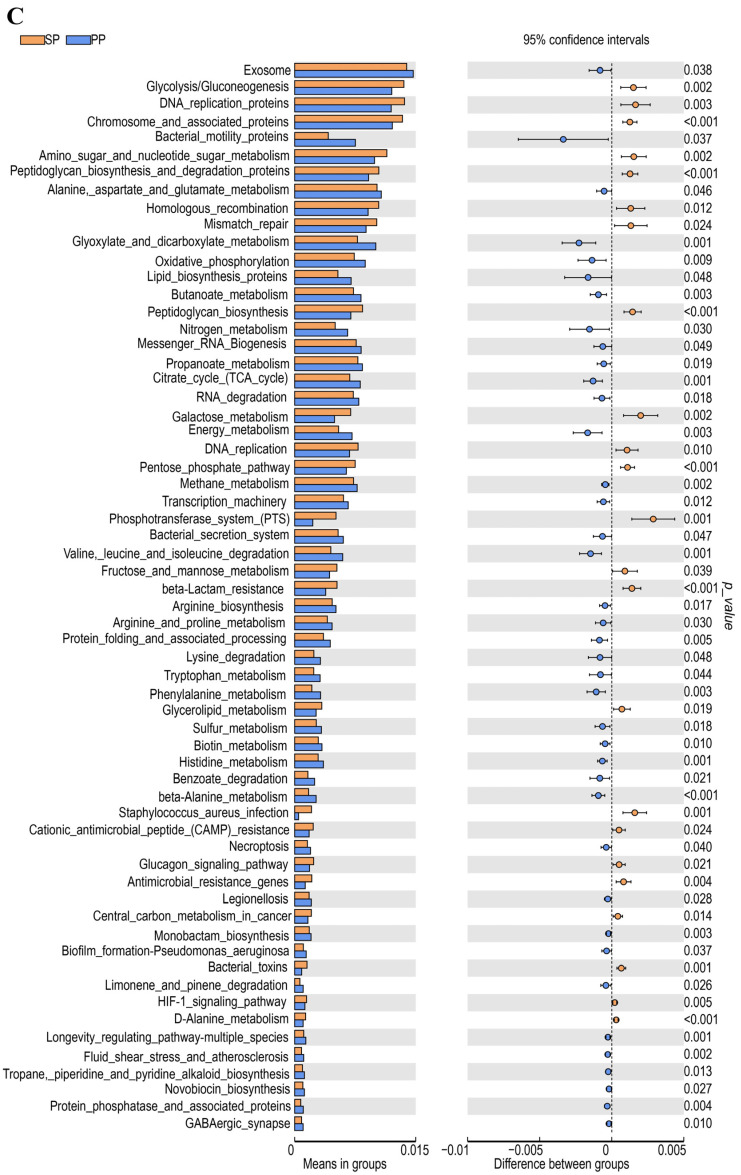
Functional prediction of Tax4Fun in feces. (**A**): Functional heatmap of fecal flora in SP and PP groups. (**B**): Functional prediction of fecal flora using Tax4Fun in SP and PP groups. (**C**): Significant *T*-test analysis of fecal flora in SP and PP groups.

The functional roles of the fecal microbiota in both groups were further investigated, and differences in their microbiota functions were identified through Tax4Fun-based functional predictions. As shown in Figure 5B, the core functions of the fecal microbiota in both groups encompass metabolism, genetic information processing, cellular processes, unclassified functions, human diseases, and organismal systems. Notably, metabolism, genetic information processing, and cellular processes collectively account for over 80% of the total functions, underscoring their critical roles in gut microbiota activity.

At the Level 3 functional analysis (Figure 5C), significant differences (*p* < 0.01) were observed between the SP and PP groups across several key metabolic and biosynthetic pathways.

### 3.2. Metabolomics Analysis Results

#### 3.2.1. Correlation Analysis and PCA of QC Samples Based on Relative Quantification of Metabolites

**Figure 6:**
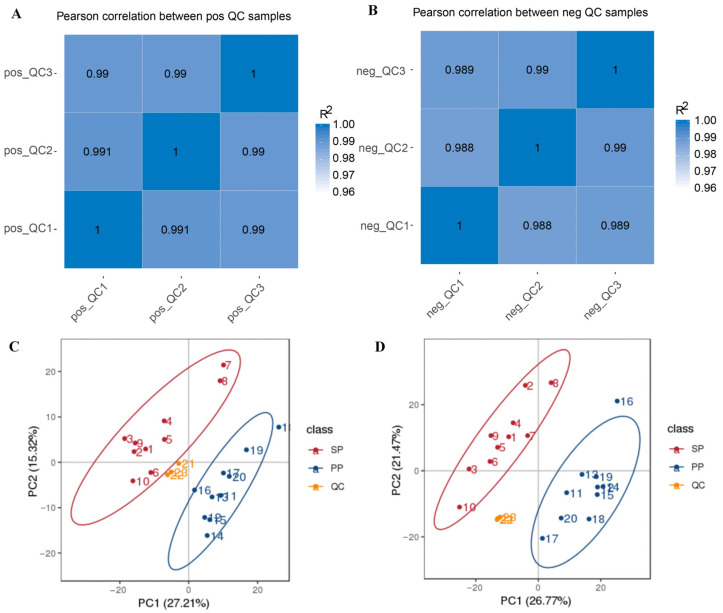
Correlation analysis and PCA of QC samples. (**A**): Correlation analysis of QC samples in positive ion mode. (**B**): Correlation analysis of QC samples in negative ion mode. (**C**): PCA of samples and QC samples in positive ion mode based on relative metabolite quantification. (**D**): PCA of samples and QC samples in negative ion mode based on relative metabolite quantification.

To further investigate the complex relationship between the gut microbiota and metabolites in the SP and PP groups, an integrated analysis combining 16S rRNA sequencing and metabolomics was conducted. The correlation of the QC samples with the PCA results provided essential quality control metrics. As shown in Figure 6A,B, the correlation of the QC samples exceeded 0.99 in positive ion mode and 0.98 in negative ion mode. Additionally, in both ion modes, the QC samples formed tight clusters, as depicted in Figure 6C,D, indicating minimal variance among them. These results confirm that the LC-MS-based metabolite detection in this study demonstrates outstanding stability and data quality, ensuring robustness for subsequent analyses.

#### 3.2.2. Classification of Fecal Metabolites in SP Group and PP Group

**Figure 7:**
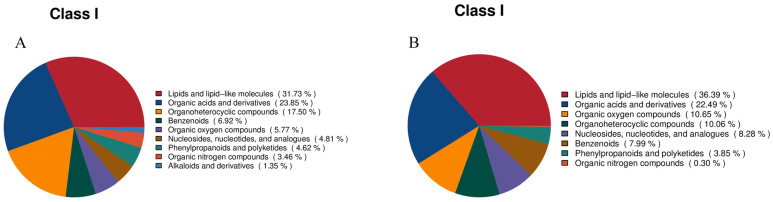
Classification of fecal metabolites. (**A**): Classification of metabolites in feces in positive ion mode. (**B**): Classification of metabolites in feces in negative ion mode.

A statistical analysis of the chemical classification of the identified metabolites is shown in Figure 7, which categorizes and quantifies the metabolites within each group. In positive ion mode (Figure 7A), the metabolites are classified into nine categories. Lipids and lipid-like molecules are the dominant group, comprising 37.54%, highlighting the prominence of lipid metabolism in the intestinal microecology of both squabs and parent pigeons. Organic acids and derivatives, critical for energy metabolism and material cycling, account for 23.85%. Organoheterocyclic compounds represent 17.50%, followed by benzenoids at 6.92%, organic oxygen compounds at 5.77%, and nucleosides, nucleotides, and analogs at 4.81%. Phenylpropanoids and polyketides contribute 4.62%, organic nitrogen compounds make up 3.46%, and alkaloids and derivatives account for 1.35%. In total, these categories represent the metabolite composition of both squabs and parent pigeons. In negative ion mode (Figure 7B), the metabolites are classified into eight categories. Lipids and lipid-like molecules remain the largest group at 36.39%, with organic acids and derivatives comprising 22.49%. Organic oxygen compounds constitute 10.65% and organoheterocyclic compounds represent 10.06%. Nucleosides, nucleotides, and analogs account for 8.28%, benzenoids for 7.99%, phenylpropanoids and polyketides for 3.85%, and organic nitrogen compounds are significantly reduced to 0.30%. These shifts in the metabolite distribution between ion modes suggest that different detection conditions may influence intestinal microecology, reflecting its complex and dynamic nature.

#### 3.2.3. Screening Results of Different Metabolites in Feces of SP Group andPP Group

Table 2 summarizes the differential metabolite analysis of the feces of the SP and PP groups. In cation mode, 796 metabolites were identified. The PLS-DA model was applied to screen for potential biomarkers based on VIP > 1, *p* < 0.05, and FC ≥ 2 or FC ≤ 0.5. This approach identified 218 differential metabolites between the SP and PP groups, with 139 upregulated and 79 downregulated. In anion mode, 462 metabolites were detected, and the same criteria revealed 148 differential metabolites, including 98 upregulated and 50 downregulated.

#### 3.2.4. Volcanic Map of Differential Metabolites in Feces of SP Group and PP Group

**Figure 8:**
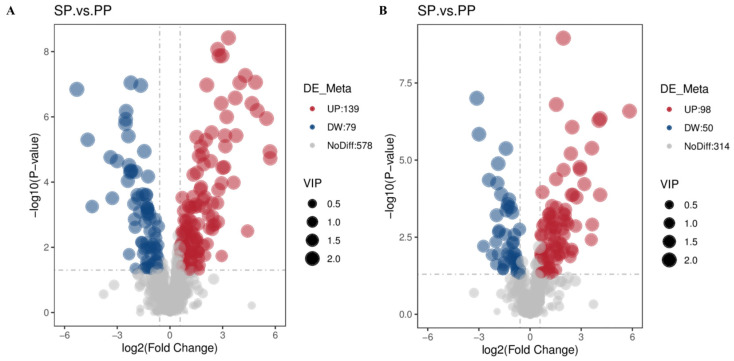
Volcano plot of the differential metabolites in the feces. (**A**): Volcano plot of differential metabolites in the feces of the SP and PP groups in positive ion mode. (**B**): Volcano plot of differential metabolites in the feces of the SP and PP groups in negative ion mode.

The volcano plot provides a clear visualization of the distribution of differential metabolites. The x-axis represents the fold change (log2) of the metabolites between the groups, while the y-axis indicates the significance level (−log10 (*p*-value)). Each point on the plot corresponds to a metabolite, with significantly upregulated metabolites shown as red dots, significantly downregulated metabolites as blue dots, and the dot size reflecting the VIP value. Figure 8A displays the differential metabolic volcano plots for positive ion mode, and Figure 8B shows those for negative ion mode.

#### 3.2.5. Correlation Analysis of Different Metabolites in Feces of SP Group and PP Group

**Figure 9:**
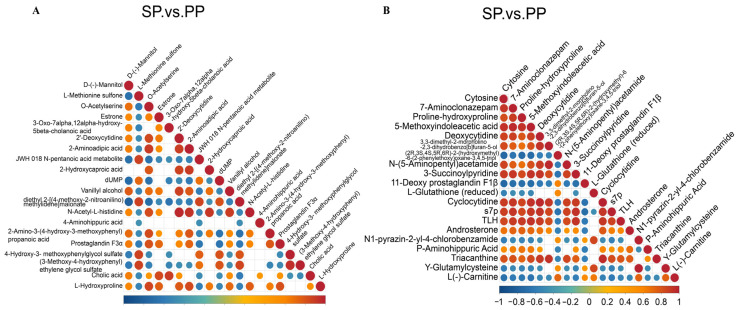
Correlation analysis of the differential metabolites in the feces. (**A**): Correlation analysis of differential metabolites in the feces of the SP and PP groups in positive ion mode. (**B**): Correlation analysis of differential metabolites in the feces of the SP and PP groups in negative ion mode.

To explore the intrinsic relationships between the differential metabolites, a correlation analysis was performed. Metabolites can exhibit either synergistic or antagonistic interactions; those with similar trends in change are positively correlated, while opposing trends indicate negative correlations. This analysis aimed to assess the consistency of the changes among metabolites by calculating the Pearson correlation coefficient for each metabolite pair. A coefficient near 1 signifies a strong positive correlation, while a value close to −1 indicates a strong negative correlation. Statistical significance was defined as *p* < 0.05. As shown in Figure 9, significant correlation patterns were observed for differential metabolites in both the positive and negative ion modes. In positive ion mode (Figure 9A), numerous metabolites exhibited strong positive correlations, suggesting the potential collaborative regulation of key biological processes. A similar pattern of positive correlations was found in negative ion mode (Figure 9B), further confirming the close functional interactions between the metabolites.

#### 3.2.6. KEGG Classification of Differential Metabolites in Feces of SP Group and PP Group

**Figure 10:**
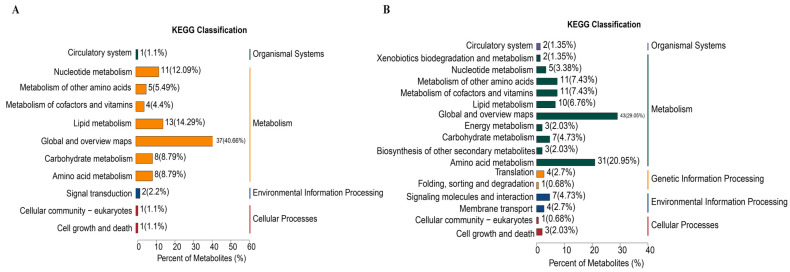
KEGG classification of differential metabolites in feces. (**A**): Pathway annotation for KEGG in positive ion mode. (**B**): Pathway annotation for KEGG in negative ion mode.

Based on the metabolite classification data from the KEGG database, a statistical analysis of the annotated differential metabolites was performed, and the results are presented visually. Figure 10A shows that in positive ion mode, the differential metabolites are classified into five categories: organismal systems, metabolism, genetic information processing, environmental information processing, and cellular processes. Specifically, 2 metabolites are associated with organismal systems, 126 with metabolism, 5 with genetic information processing, 11 with environmental information processing, and 4 with cellular processes. Figure 10B displays the distribution of differential metabolites in negative ion mode across four categories: organismal systems, metabolism, environmental information processing, and cellular processes. This includes 1 metabolite in organismal systems, 86 in metabolism, 2 in environmental information processing, and 2 in cellular processes. These results are consistent with the Tax4Fun functional annotation data from the feces of the squabs and parent pigeons, further reinforcing the strong connection between their gut microbiota and metabolites.

#### 3.2.7. KEGG Enrichment Pathway Analysis of Differential Metabolites in Feces of SP Group and PP Group

**Figure 11:**
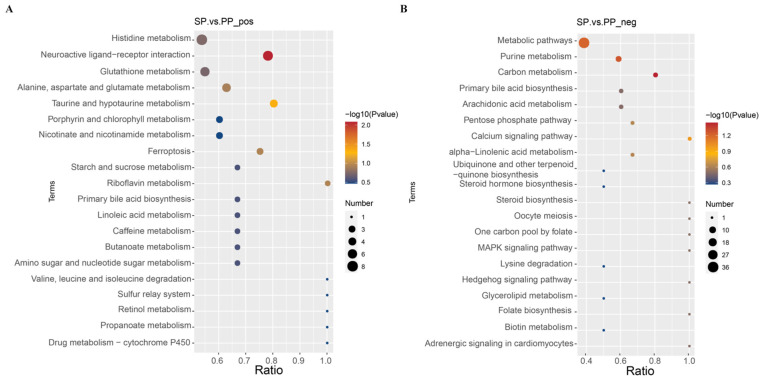
KEGG enrichment pathway analysis of the differential metabolites in the feces. (**A**): Enrichment results of KEGG in the feces of the SP and PP groups in positive ion mode. (**B**): Enrichment results of KEGG in the feces of the SP and PP groups in negative ion mode.

An enrichment analysis of the KEGG-annotated differential metabolites was conducted using the clusterProfiler package and a hypergeometric test. The resulting enrichment dot plot, shown in Figure 11, illustrates that smaller *p*-values indicate more significant enrichment. In positive ion mode, the differential metabolites are predominantly enriched in pathways such as Histidine metabolism; Neuroactive ligand–receptor interaction, Glutathione metabolism; Alanine, Aspartate, and Glutamate metabolism; and Taurine and hypotaurine metabolism. These pathways are central to amino acid metabolism, neuroregulation, antioxidant defense, and other critical functions, underscoring the significant impact of gut microbiota on host metabolism and health. In negative ion mode, the differential metabolites are mainly enriched in pathways related to Metabolism, Purine metabolism, and Carbon metabolism, which are essential for energy metabolism and material cycling. This highlights the gut microbiota’s critical role in maintaining the host’s energy balance and material utilization.

#### 3.2.8. Results of Correlation Analysis of Different Metabolites in Feces of SP Group and PP Group

**Figure 12:**
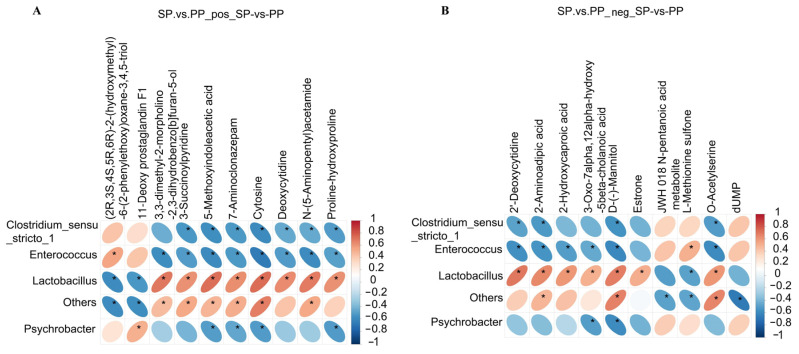
Correlation analysis of the differential metabolites in the feces. (**A**): Correlation analysis results of the differential metabolites in the feces of the SP and PP groups in positive ion mode. (**B**): Correlation analysis results of the differential metabolites in the feces of the SP and PP groups in negative ion mode. An asterisk (*) indicates significant relevance in the correlation analysis.

As shown in Figure 12, *Lactobacillus* exhibits a significant positive correlation with eight metabolites, including 3,3-dimethyl-2-morpholino-2,3-dihydrobenzo[b]furan-5-ol, 3-Succinoylpyridine, 5-Methoxyindoleacetic acid, 7-Aminoclonazepam, Cytosine, Deoxycytidine, *N*-(5-Aminopentyl)acetamide, and Proline–hydroxyproline. In contrast, both Clostridium_sensu_stricto_1 and *Enterococcus* show significant negative correlations with seven metabolites: 3-Succinoylpyridine, 5-Methoxyindoleacetic acid, 7-Aminoclonazepam, Cytosine, Deoxycytidine, N-(5-Aminopentyl)acetamide, and Proline–hydroxyproline. Additionally, Clostridium_sensu_stricto_1 demonstrates a significant negative correlation with 3,3-dimethyl-2-morpholino-2,3-dihydrobenzo[b]furan-5-ol, further illustrating the complex interactions between these bacteria and metabolites.

## 4. Discussion

The Tarim pigeon, a domesticated breed developed through long-term selective breeding by residents along the Yarkand and Tarim Rivers in the Western Tarim Basin, descends from the Xinjiang subspecies of the rock pigeon. Officially included in China’s National Livestock and Poultry Genetic Resources Inventory on 15 January 2010, it is also listed in the “China Livestock and Poultry Genetic Resources”. Recognized for its resilience, adaptability, and disease resistance, the Tarim pigeon holds substantial potential for development and utilization. Traditionally, it has been raised through free-range farming, preserving its wild characteristics. However, as the pigeon industry scales and intensifies, enclosed group and cage farming methods have become more common. Due to its unique physiological traits, such as pair production and parental feeding, managing Tarim pigeons requires different practices compared to conventional poultry. Presently, feeding management strategies remain underdeveloped. In intensive farming systems, the limited sources of intestinal microorganisms for squab pigeons could negatively affect their health, particularly in confined environments.

### 4.1. Microbial Composition and Diversity in Squabs and Parent Pigeons

Intestinal microorganisms play a critical role in overall health, particularly during early colonization in young animals, influencing growth, intestinal health, and immune function maturation. Unlike chickens, ducks, and geese, squabs primarily obtain their early nutrition from pigeon milk. During this period, they receive not only essential nutrients but also microorganisms from their parents’ gastrointestinal tract. Pigeon milk contains a high concentration of microorganisms, which, as squabs are fed, are transferred from the parent pigeons’ crop to the squabs, impacting the development of their intestinal microflora [17]. Studies indicate that the microbial diversity in pigeons’ crops exceeds that in the small intestine and rectum, with lactic acid bacteria predominating in the intestines of healthy 21-day-old squabs. However, infections such as gallinae reduce the microbial community’s richness and diversity in squab pigeon milk. Similarly, Poroyko’s research demonstrated that mammalian milk contains specific prebiotics that significantly alter the intestinal microbiota in lactating infants [18]. Microorganisms entering the crop help maintain intestinal flora balance and secrete essential nutrients vital for pigeon growth. Gillespie et al. [19] demonstrated that substituting pigeon milk for chick diets still allowed bacteria from the pigeon milk to be detected in the chicks’ cecum. Furthermore, supplementing probiotics during the breeding period has been shown to support the development of healthy microflora in squabs, enabling them to adapt to more complex environments [20]. Therefore, the species and abundance of gastrointestinal microorganisms are likely key factors in the early colonization of the intestinal microflora in squabs. Understanding the composition, classification, and diversity of intestinal microflora in both parent pigeons and squabs is essential for shaping early microbial colonization through the regulation of the parent pigeons’ gut flora. This research provides valuable insights for early intervention in the intestinal microbial homeostasis in squabs through the comparison and analysis of fecal bacterial diversity and metabolites in both squabs and parent pigeons.

High-throughput sequencing revealed 782 and 1440 OTUs in the feces of squabs and parent pigeons, respectively. A Venn diagram analysis showed that 251 OTUs were shared, 531 were specific to the squabs, and 1189 were specific to the parent pigeons, indicating a higher bacterial load in the latter. Increased intestinal microbial diversity generally correlates with better microecosystem balance and enhanced gut health [21,22]. An α-diversity analysis demonstrated that the parent pigeons’ feces contained 1440 OTUs, with a Chao1 index of 241.16, a Shannon index of 3.76, and a Simpson index of 0.80. In contrast, squabs exhibited 782 OTUs, a Chao1 index of 140.75, a Shannon index of 3.44, and a Simpson index of 0.79. The significantly higher bacterial diversity and abundance in parent pigeons suggest a more complex and healthier microbiome in contrast with the simpler structure and composition in squabs, which is consistent with Han Pengmin’s findings in 12-day-old white feather pigeons, where a reduced microbial diversity increased susceptibility to pathogenic infections [23]. A β-diversity analysis revealed distinct bacterial profiles between squabs and parent pigeons, with pigeon milk at 12 days showing 2181 OTUs, a Chao1 index of 923.84, a Shannon index of 6.01, and a Simpson index of 0.93. The squabs’ intestinal contents at this stage contained 1396 OTUs, a Chao1 index of 1173.95, a Shannon index of 7.03, and a Simpson index of 0.95. After 28 days, the squabs exhibited 790 OTUs, a Chao1 index of 506.83, a Shannon index of 4.14, and a Simpson index of 0.73. In conclusion, compared to the pigeon milk, the species richness and diversity of the gut bacteria in squabs were significantly lower, with both decreasing as squabs aged. This decline is likely attributed to the transition away from pigeon milk as the primary dietary source during maturation [24].

Research has shown that under normal conditions, beneficial bacteria such as *Lactobacillus*, *Aeriscardovia*, and *Bifidobacterium* colonize the intestines of squabs, playing a pivotal role in promoting intestinal peristalsis and enhancing nutrient digestion and absorption [25,26]. This microbial presence is particularly important for the rapid weight gain observed during squabs’ growth and development. The intestinal microbiota of squabs is strongly influenced by pigeon milk. Ding et al. [20] reported that Firmicutes, Proteobacteria, and Actinobacteria are common to both the gut and milk microbiota, whereas Bacteroidetes and Cyanobacteria are more abundant in pigeon milk. In contrast, Tenericutes predominates in the pigeon gut. At the genus level, 12 genera differed significantly between the pigeon milk and gut microbiota (*p* < 0.05). The dominant genera in pigeon milk included *Lactobacillus* (42%), *Enterococcus* (9%), *Veillonella* (9%), and *Bifidobacterium* (8%), while the pigeon gut was dominated by *Turicibacter* (20%), *Lactobacillus* (13%), *Unclassified_Clostridiaceae* (12%), *Enterococcus* (12%), and *Unclassified_Mollicutes* (9%). Notably, *Gallibacterium*, *Veillonella*, and *Lactobacillus* were nearly seven-, four-, and two-fold more abundant in pigeon milk than in the gut. In this study, Firmicutes, Cyanobacteria, Actinobacteria, Proteobacteria, and Bacteroidetes were among the top ten bacterial phyla in both the squab and parent pigeon feces, with Firmicutes being the dominant phylum. These results align with those of Eiling Chen, who also identified Firmicutes, Proteobacteria, and Actinobacteria as the dominant phyla in the ileum of squabs, with Firmicutes comprising over 90% of the microbial community. Due to its strong fermentation capabilities and production of short-chain fatty acids (SCFAs), Firmicutes is essential for nutrient absorption, intestinal health, and host energy metabolism. During the rapid growth phase, the high abundance of Firmicutes in squabs enhances nutrient utilization and fat deposition, contributing to their swift development. Moreover, the substantial presence of Firmicutes in parent pigeon feces suggests potential transmission to squabs through direct contact or pigeon milk, playing a key role in establishing the intestinal microbiota in squabs. This is consistent with Ding et al.’s [27] findings, which confirmed the presence of a significant microbial population in pigeon milk capable of being transmitted from parents to squabs.

In this study, the abundance of Proteobacteria in the squabs was significantly higher than in the parent pigeons, suggesting that the squabs’ intestines may be more susceptible to harmful bacteria. This highlights the importance of monitoring the impact of pathogenic bacteria on intestinal health during the early squab stage [28]. Ma et al. [29] reported that at the phylum level, Firmicutes and Proteobacteria were the predominant bacteria in the colons of squabs, together accounting for over 93% of the total bacterial community. Additionally, *Enterococci* and *Corynebacterium* were abundant in squab feces, with *Enterococci*, *Lactobacillus*, and *Corynebacterium* being particularly prevalent. Specifically, *Enterococcus cecorum* and *Lactobacillus ingluviei* were significantly more abundant in squabs compared to parent pigeons. In contrast, parent pigeon feces exhibited significantly higher levels of Lactobacillaceae, *Clostridium*, *Lactobacillus*, and Candidatus Arthromitus, along with species-level *Lactobacillus panis* and *Lactobacillus coleohominis*. These findings align with Zhao et al. [28], who also identified *Lactobacillus* and *Enterococcus* as the dominant bacteria in pigeon intestines. Although *Enterococci* are part of the normal gastrointestinal flora in mammals and birds, they can act as opportunistic pathogens [30]. For instance, *Enterococcus faecalis* is associated with amyloid arthropathy in poultry and pulmonary hypertension syndrome in broilers [31], while *Enterococcus cecorum* is a common cause of increased first-week mortality in chicks and has been linked to hepatic granulomas in turkey poults [21]. The significantly higher abundance of *Enterococcus cecorum* in squabs indicates their heightened susceptibility to infection. Therefore, careful monitoring of the intestinal microbiota during the lactation stage is essential for the development of strategies to prevent gastrointestinal diseases. *Lactobacillus ingluviei*, a species found in the gastrointestinal tracts of pigeons and ostriches, has been shown to influence body weight gain. Increased levels of *Lactobacillus ingluviei* in the intestinal microbiome can significantly improve weight gain and alter metabolism, potentially contributing to squabs reaching a body weight of 500 g by 28 days. A Lefse analysis further identified 20 distinct bacterial species in the fecal flora of squabs and parent pigeons, with 14 species present in the parent pigeons and 6 in the squabs. Notable species included Lactobacillaceae, *Lactobacillus*, Candidatus Arthromitus, Clostridiaceae, and various genera within the Clostridia order.

Candidatus Arthromitus plays a critical role in maintaining intestinal innate immune function and supporting the development of the acquired immune system. A reduction in its abundance can lead to intestinal immune imbalance and trigger inflammatory responses [26]. In this study, the abundance of Candidatus Arthromitus in squabs was significantly higher than in parent pigeons, suggesting that squabs acquire innate immunity early via pigeon milk. Clostridiaceae, often associated with poor health outcomes, was also found in higher abundance in squab feces, indicating that the squab gastrointestinal tract is more vulnerable to infections by harmful bacteria, highlighting increased susceptibility in this species. Despite natural birth, early-life environmental exposures appear to have a limited impact on the gut ecosystem. The Tax4Fun functional annotation of fecal samples from both squabs and parent pigeons identified seven major gene functions at Level 1. Metabolism, genetic information processing, and environmental information processing genes exhibited the highest functional abundance, though no significant differences were observed in the functional gene abundances between squab and parent pigeon fecal bacteria. A further Level 3 functional analysis revealed 303 functional genes, 62 of which showed significant differences. Among these, 23 functional genes were significantly or highly significantly more expressed in squab fecal bacteria than in parent pigeons. Notably, one key pathway identified was that of bacterial motility proteins, which are essential for pathogenic bacteria to adhere to villi and invade epithelial cells, potentially causing intestinal inflammation [32,33]. This finding aligns with the observed higher abundance of *Enterococcus cecorum* in the squab feces compared to the parent pigeons.

Both bacterial motility proteins and the bacterial secretion system were prominently expressed in the feces of both squabs and parent pigeons. According to Ó Cróinín et al. [34], motility proteins are critical for bacterial attachment to epithelial cells and movement toward or away from stimuli. The bacterial secretion system, classified into Types I-IV, facilitates the active transport of proteins from the cytoplasm to the bacterial surface [35]. These systems are essential for gut colonization, enabling bacterial invasion of the mucosal surface and closely interacting with flagella assembly and motility proteins. Interestingly, both bacterial motility and secretion systems are essential for host adhesion, infection, and colonization, mediated by genes responsible for the biosynthesis of fimbriae, flagella, outer membranes, metabolic processes, and lipopolysaccharides [36,37].

### 4.2. Metabolomic Profiles and Functional Pathway Analysis

The intestinal microbiota and their metabolites are critical for maintaining intestinal homeostasis, which directly impacts the host’s overall health. A significant proportion of the metabolites synthesized by the microbiota are absorbed into the host, playing a key role in health maintenance. Some of these metabolites possess functional properties that support growth, development, and health [38]. In this study, nine major metabolite categories were identified in the feces of pigeon squabs and their parents, with lipids and lipid-like molecules constituting 31.73% and organic acids and derivatives 23.85% of the total metabolites. Under cationic mode, 796 differentially expressed metabolites were detected in the feces of both squabs and parent pigeons, of which 218 exhibited significant differences: 139 were upregulated and 79 downregulated. A KEGG enrichment analysis revealed that these metabolites primarily participate in five functional categories: organismal systems, metabolism, genetic information processing, environmental information processing, and cellular processes. Metabolism emerged as the dominant functional category, encompassing pathways involved in amino acid metabolism, the biosynthesis of secondary metabolites, carbohydrate metabolism, energy metabolism, lipid metabolism, the metabolism of cofactors and vitamins, nucleotide metabolism, and xenobiotic biodegradation. Notably, 43 differentially expressed metabolites were linked to the “Global and overview maps” function, and 31 were associated with amino acid metabolism. A further KEGG pathway enrichment analysis highlighted 33 enriched metabolic pathways, with two pathways—Neuroactive ligand–receptor interaction (*p* = 0.0083) and Taurine and hypotaurine metabolism (*p* = 0.0474)—showing particular significance. In the Neuroactive ligand–receptor interaction pathway, seven metabolites were enriched, including hydrocortisone (upregulated), adenosine glycoside (downregulated), morphine (upregulated), taurine (downregulated), histamine (upregulated), serotonin (upregulated), and 2-arachidonoyl glycerol (upregulated). The Taurine and hypotaurine metabolism pathway enriched four metabolites: hypotaurine (upregulated), taurocholic acid (upregulated), L-glutamate (upregulated), and taurine (downregulated).

The Neuroactive ligand–receptor interaction pathway is essential for various biological processes. Research indicates that its upregulation can alleviate disturbances in amino acid metabolism, contributing to metabolic balance regulation [39]. Moreover, this pathway is essential in managing arrhythmias and depression, and it plays a critical role in early embryonic development by supporting healthy embryo growth. In this study, the Neuroactive ligand–receptor interaction pathway emerged as a significantly enriched pathway for differential metabolites in squab feces, highlighting the importance of metabolites such as hydrocortisone, adenosine, morphine, taurine, histamine, serotonin, and 2-arachidonoyl glycerol in early squab development, consistent with their known biological roles. Additionally, the Taurine and hypotaurine metabolism pathway, recognized for its antibacterial properties and involvement in host resistance to pathogens, aligns with the biological functions of key metabolites, including hypotaurine, taurocholic acid, L-glutamate, and taurine [40]. Hong et al. [41] demonstrated that Taurine and hypotaurine metabolism can mitigate liver injury in mice, emphasizing its protective role in organ health. Similarly, Yu et al. [42] identified three amino acid metabolic pathways—Arginine biosynthesis, Taurine and hypotaurine metabolism, and Alanine, Aspartate, and Glutamate metabolism—as significant in both childhood and adult obesity studies, highlighting the fundamental role of amino acid metabolism in various physiological conditions.

Taurine, a sulfur-containing non-protein amino acid found in animal-based foods, exhibits a range of biological functions, including antioxidant activity, immunomodulation, anti-apoptotic effects, and bile synthesis promotion. Its derivative, hypotaurine (HTU), also displays notable antioxidant properties. Taurine functions as a central inhibitory neurotransmitter or modulator, regulating neural excitability [43]. Furthermore, it combines with bile acids in the liver to form taurocholic acid, which aids in fat emulsification, enhances lipase activity, and facilitates the digestion and absorption of fats and fat-soluble substances. Taurine also improves intestinal mucosa structure, stimulates digestive enzyme activity, and promotes nutrient absorption. Studies have shown that taurine alleviates oxidative stress-induced damage in tissues, organs, or cells from various stressors, such as high-fat diets, heavy metal exposure (e.g., lead and cadmium), excessive manganese, carbon tetrachloride, diquat, paraquat, Streptococcus uberis, lipopolysaccharides, and intense exercise [44,45]. In this study, four differentially expressed metabolites were enriched in the Taurine and hypotaurine metabolism pathway, with their effects consistent with previous findings. This suggests that metabolites produced by the gut microbiota in pigeon squabs play a beneficial role in promoting early health, supporting growth, and enhancing resilience during development.

A Pearson correlation analysis was performed and a heatmap was generated to visualize the associations between significantly different bacterial genera and differentially expressed metabolites identified via metabolomics. This analysis aimed to explore the relationship between the gut microbiota diversity and metabolite profiles in the samples. The correlation heatmap revealed significant positive and negative correlations between four bacterial genera—*Lactobacillus*, *Enterococcus*, *Psychrobacter*, and Clostridium_sensu_stricto_1—and ten metabolites, including (2*R*,3*S*,4*S*,5*R*,6*R*)-2-(hydroxymethyl)-6-(2-phenylethoxy)oxane-3,4,5-triol, 11-Deoxy prostaglandin F1, and 3,3-dimethyl-2-morpholino-2,3-dihydrobenzo[b]furan-5-ol. *Lactobacillus* exhibited a strong positive correlation with eight metabolites, including 3,3-dimethyl-2-morpholino-2,3-dihydrobenzo[b]furan-5-ol, 3-Succinoylpyridine, 5-Methoxyindoleacetic acid, 7-Aminoclonazepam, Cytosine, Deoxycytidine, *N*-(5-Aminopentyl)acetamide, and Proline–hydroxyproline, indicating its role in upregulating these metabolites.

Lactic acid bacteria (LAB), a key component of the gut microbiota, initiate colonization shortly after birth and participate in a range of metabolic processes. Their critical characteristics include attachment to the intestinal mucosa, the establishment of stable colonization, and significant metabolic and enzymatic activity. LAB produce a variety of bioactive metabolites, such as cytosine, which can inhibit the carbon, nitrogen, and phosphorus metabolism of Candida albicans, thereby limiting its growth and improving gut health. Furthermore, LAB metabolism is closely linked to the production of SCFAs and taurine, which are crucial in regulating intestinal barrier function, enhancing immune tolerance, and maintaining intestinal homeostasis. For example, SCFAs, including acetate, propionate, and butyrate, inhibit pathogenic colonization by lowering intestinal pH, improve intestinal barrier function, and bolster anti-inflammatory responses and immune system performance [46]. Butyrate, a key molecule in intestinal barrier repair and immune regulation, activates G protein-coupled receptors (GPRs) and inhibits histone deacetylases (HDACs), significantly alleviating intestinal inflammation and enhancing tight junction protein functionality [47]. Taurine, another important metabolite derived from LAB, regulates bile acid metabolism, reduces intestinal inflammation, and supports host growth and disease prevention [48].

The health benefits of these metabolites have been further substantiated by studies involving probiotics isolated from poultry gut and feces. Research focusing on the direct screening of LAB from the gastrointestinal tracts and feces of poultry species for potential reapplication has demonstrated significant effects. LAB and Bifidobacterium strains isolated from the intestines or feces of chickens, ducks, geese, and pigeons not only enhance gut microbial composition but also notably improve immune function and promote growth performance through their metabolic activities. For instance, when LAB and Bifidobacterium strains isolated from duck guts were used as feed additives, they substantially increased beneficial bacteria, reduced pathogenic bacteria such as *Escherichia coli*, and enhanced weight gain and feed conversion efficiency [49]. Similarly, strains of *Clostridium* and *Bacillus subtilis* from goose guts improved gut microbial diversity, boosted antioxidant capacity, and strengthened the intestinal barrier, ultimately leading to better growth performance [50].

In pigeons, supplementation with LAB-based probiotics derived from their feces significantly increased the relative abundance of Lactobacillus, optimizing gut microbial composition. However, these benefits diminished rapidly upon discontinuation, underscoring the importance of continuous supplementation for maintaining gut microbiota balance [51]. Additionally, Prentza et al. demonstrated that the combined use of probiotics and prebiotics significantly enhanced gut barrier function, optimized microbial diversity, and improved growth performance in chickens [52].

This “host-derived for host use” precision probiotic development strategy not only enhances the adaptation and colonization efficiency of probiotics but also addresses the inefficiencies often encountered with cross-species applications. By modulating microbial communities and their metabolites, this approach significantly bolsters the host’s immune system and metabolic networks. It offers precise and sustainable solutions for poultry health management while providing robust theoretical support for metabolomics-based probiotic applications. Moreover, it paves the way for practical applications in poultry farming. A deeper understanding of the interplay between the microbiota and metabolites in pigeon and squab feces can reveal further functional diversity in microbes and drive innovative probiotic applications in sustainable farming.

## 5. Conclusions

In conclusion, 16S rRNA sequencing revealed significant differences in both the α and β diversity of fecal bacteria between squabs and parent pigeons. Although the bacterial species were largely similar, their relative abundances varied considerably. A metabolite analysis using LC-MS/MS indicated a higher abundance of metabolites in parent pigeon feces. These findings suggest that the modulation of the gut microbiota with beneficial bacteria, such as probiotics, may offer potential health benefits to the host by influencing microbial composition and metabolite profiles.

## Figures and Tables

**Table 1:** Analysis of bacterial Alpha diversity in feces.

Item	PP Group	SP Group	SEM	*p*-Value
chao1	241.16 ^a^	140.75 ^b^	35.19	0.02
dominance	0.20	0.23	0.06	0.67
goods_coverage	1.00	1.00	0.00	0.95
observed_otus	240.90 ^a^	140.30 ^b^	35.10	0.02
pielou_e	0.48	0.48	0.04	0.97
shannon	3.76	3.44	0.35	0.37
simpson	0.80	0.77	0.06	0.67

No letter or the same lowercase letter in the peer data indicates no significant difference (*p* > 0.05), whereas different lowercase letters denote a significant difference (*p* < 0.05), as illustrated in the Table above.

**Table 2:** Screening results of different metabolites in feces of SP and PP groups.

Compared Samples	Num. of Total Ldent.	Num. of Total Sig.	Num. of Sig.up	Num. of Sig.down
SP. vs. PP_pos	796	218	139	79
SP. vs. PP_ng	462	148	98	50

## Data Availability

The data supporting the findings of this study are available from the corresponding author upon reasonable request.
